# White Matter Hyperintensities in Young Patients from a Neurological Outpatient Clinic: Prevalence, Risk Factors, and Correlation with Enlarged Perivascular Spaces

**DOI:** 10.3390/jpm13030525

**Published:** 2023-03-15

**Authors:** Qiaoqiao Zou, Mingliang Wang, Danni Zhang, Xiaoer Wei, Wenbin Li

**Affiliations:** Department of Radiology, Shanghai Sixth People’s Hospital Affiliated to Shanghai Jiao Tong University School of Medicine, Shanghai 200233, China

**Keywords:** white matter hyperintensities, enlarged perivascular spaces, young patients, MRI

## Abstract

(1) Background: to investigate the prevalence of white matter hyperintensities (WMH), risk factors, and correlation with enlarged perivascular spaces (ePVS) among young patients (age, 16–45 years) in a neurological outpatient clinic. (2) Methods: a total of 887 young patients who underwent a head magnetic resonance imaging (MRI)examination between 1 June 2021, and 30 November 2021, were included in this study. Paraventricular WMH (PWMH), deep WMH (DWMH), ePVS in the centrum semiovale (CSO-ePVS), and basal ganglia (BG-ePVS) were rated. Logistic regression analysis was used to identify the best predictors for the presence of WMH and, for the association of the severity of ePVS with the presence of WMH. Goodman–Kruskal gamma test was used to assess the correlation between the severity of ePVS and WMH. (3) Results: the prevalence of WMH was 37.0%, with low severity. Age, hypertension (*p* < 0.001), headache (*p* = 0.031), syncope (*p* = 0.012), and sleep disturbance (*p* = 0.003) were associated with the presence of DWMH. Age, sex (*p* = 0.032), hypertension (*p* = 0.004) and sleep disturbance (*p* < 0.001) were associated with the presence of PWMH. The severity of CSO-ePVS was associated with the presence and the severities of DWMH. The severity of BG-ePVS was associated with the presence and severities of DWMH and PWMH. (4) Conclusions: the prevalence of WMH was 37% and mild in young patients without specific causes. Older age, female, hypertension, headache, syncope, and sleep disturbance were associated with WMH. The severity of ePVS had an impact on the presence and severity of WMH in the corresponding brain regions.

## 1. Introduction

White matter hyperintensities (WMH) are one of the manifestations of cerebral small vessel disease (CSVD), detected by magnetic resonance imaging (MRI) [1]. WMH were named after the high intensity observed on T2-weighted and T2 fluid-attenuated inversion recovery sequences (FLAIR) [2]. Several studies have reported that WMH are associated with stroke, dementia, and Alzheimer’s disease [3]. Therefore, understanding WMH prevalence, risk factors, and correlation with other CSVD is imperative.

Previous studies have shown that, WMH are associated with increasing age and hypertension [4]. However, no correlation was established between sex, diabetes, hyperlipidemia, and enlarged perivascular spaces (ePVS). Some studies have reported a correlation between WMH and ePVS in middle-aged and elderly populations [2,5]. Studies of WMH in young clinical populations (≤45-years-old) are rare. Additionally, the literature on the symptomology of WMH, especially in the young without specific diseases is limited. Therefore, determining the correlation between neurological symptoms and WMH is significant. Thus, this retrospective study aimed to investigate WMH prevalence, risk factors, and correlation with ePVS in young patients without specific diseases from a neurological outpatient clinic for an in-depth understanding of WMH.

## 2. Materials and Methods

### 2.1. Study Population

**Figure 1:**
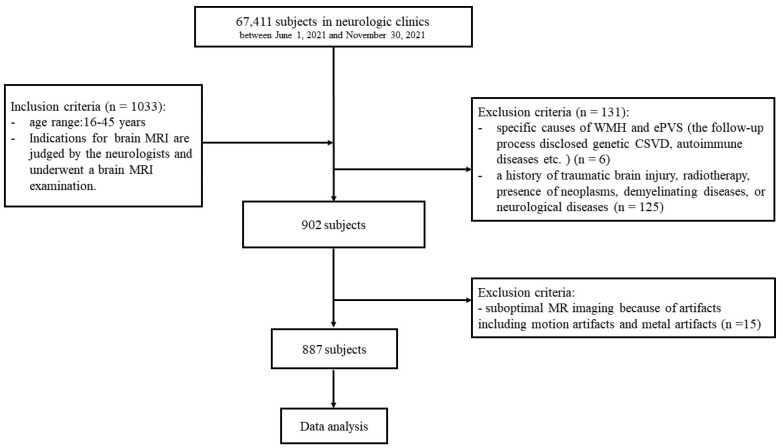
Subject screening workflow.

This retrospective study included a total of 887 outpatients (age range: 16–45 years; 389 male patients, 498 female patients) who underwent head MRI examinations between 1 June 2021, and 30 November 2021. This study did not include children (<16-years-old), as it might have shown a congenital rise in WMH [6]. The indications of MRI were determined by the neurologists and defined as follows: (1) an increase in the frequency and intensity of symptoms, such as dizziness, indicating secondary pathology; (2) any abnormal neurological finding, such as asymmetric or unilateral hearing or vision loss; (3) no neurological symptoms with brain examinations as part of the physical examination. The current study included patients with no history of a specific disease and a diagnosis of no specific disease based on head MRI results. Patients were excluded from this study if they had (1) specific causes of WMH and ePVS; (2) a history of traumatic brain injury, radiotherapy, presence of neoplasms, demyelinating diseases, or neurological diseases, or (3) suboptimal MRI because of artifacts (Figure 1).

Most of the patients (94.0%, 834/887) had neurological symptoms, including 109 patients with unspecified symptoms, whose MRI requisition forms mentioned “suspected cerebrovascular disease”. The patients with no neurological symptoms (6.0%, 53/887) underwent head MRI examinations as a part of disease screening.

Due to the retrospective nature of the study, the local institutional review board waived the informed consent requirement.

### 2.2. Data Collection

The demographic and clinical data, including age, sex, symptoms, and history of hypertension, diabetes, and hyperlipidemia, were collected from all patients.

Symptoms were determined according to the patient’s primary complaint. Symptoms found to be difficult to identify were defined as follows.

Dizziness: a feeling of sway and instability while moving or seeing, most often done while standing, sitting, or lying down.

Vertigo: a feeling (motion hallucination) characterized by episodes of the subjective illusion that one’s own body and/or outside objects are rotating or rolling in a certain direction despite being factually untrue.

Light-headedness: a continuous feeling of light-headedness or mental disorientation.

Somatic symptoms: any part of the body or organ could be affected by recurrent and changing somatic symptoms without any organic lesions being discovered by various medical examinations.

Sleep disturbance: interfering with social functioning is the subjective feeling of insufficient daytime sleep quantity and/or quality. This may involve one or more of the following: (1) prolonged sleep latency (sleep onset requiring > 30 min); (2) sleep maintenance disorder (two or more nighttime awakenings or early morning awakenings); (3) decreased sleep quality (light sleep, excessive dreaming); (4) a shortening of the total amount of time spent sleeping (usually < 6 h), or (5) residual daytime effects (dizziness, mental fatigue, drowsiness, and fatigue, on the next morning).

### 2.3. Brain MRI

All patients were scanned using the uniform 3T MRI scanning protocol (Magnetom Verio, Siemens Healthcare, Germany) that included axial T1-weighted, axial T2-weighted, axial FLAIR, axial DWI, and sagittal T1-weighted sequences.

WMH were distinguished by isointensity on T1-weighted images and high signal intensity on T2-weighted and FLAIR images [7]. According to previous experience, WMH were assessed using the Fazekas rating scale [8]. DWMH and PWMH were selected for analysis based on previous experience. PWMH were graded as follows: 0 = absent; 1 = cap; 2 = smooth halo; 3 = irregular and extending into the subcortical white matter. DWMH were graded as follows: 0 = absent; 1 = punctate foci; 2 = early-confluent, or 3 = confluent.

**Figure 2:**
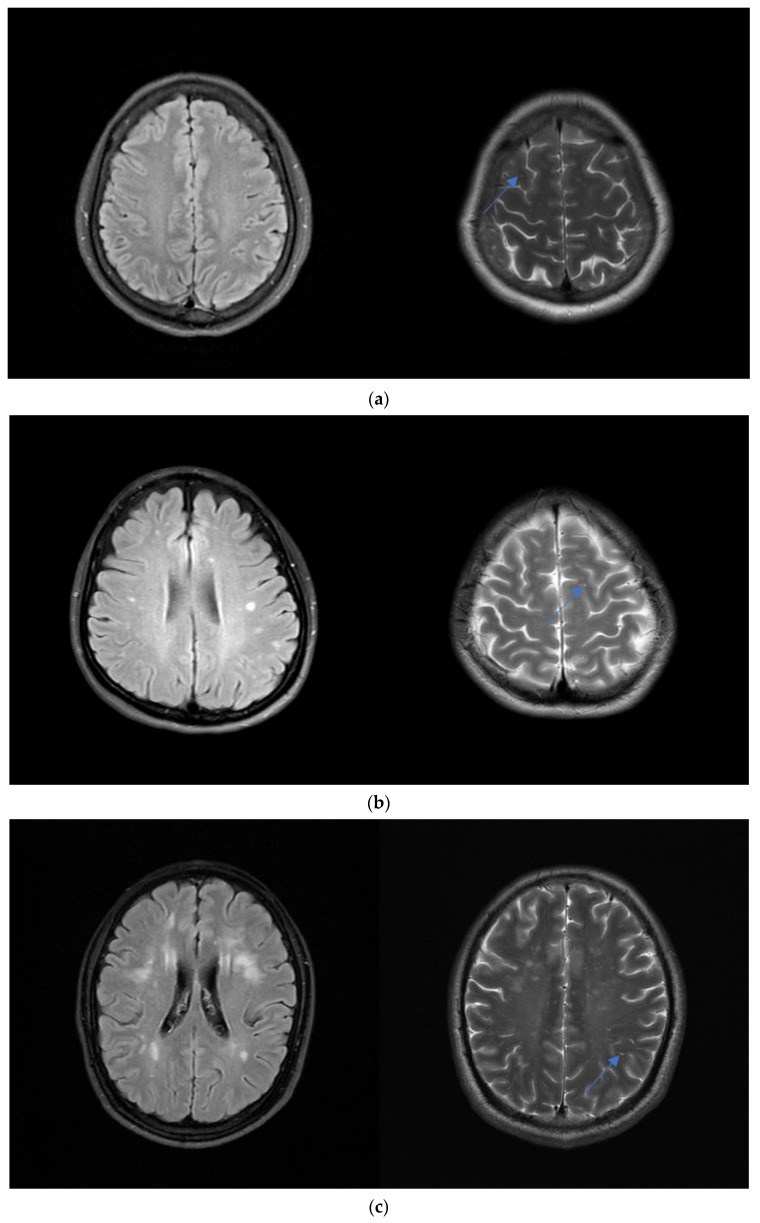
Levels of the severity of WMH. (**a**) A 23-year-old woman with a headache. Axial FLAIR MR images show mild DWMH. Axial T2-weighted images show grade 1 CSO-ePVS (blue arrow). (**b**) A 36-year-old woman with dizziness. Axial FLAIR MR images show moderate DWMH. Axial T2-weighted images show grade 2 CSO-ePVS (blue arrow). (**c**) A 45-year-old man with visual disturbance. Axial FLAIR MR images show severe DWMH. Axial T2-weighted images show grade 3 CSO-ePVS (blue arrow).

ePVS were defined as round, ovoid, or linear lesions with a CSF-like signal (hypointense on T1-weighted/FLAIR images and hyperintense on T2-weighted images) located along the penetrating arteries [7]. According to previous experience, ePVS were identified and graded at the level of centrum semiovale (CSO) and basal ganglia (BG). The level with the highest number of CSO-ePVS was selected and graded as follows: 0: no ePVS; 1: ≤10 ePVS; 2: 11–20 ePVS; 3: 21–40 ePVS; or 4: > 40 ePVS, or an uncountable number [9]. The level with the highest number of BG-ePVS was selected and graded as follows: (1) 1: <5 ePVS; (2) 2: 5–10 ePVS; and (3) 3: 10–20 ePVS; (4) 4: > 20 ePVS [9]. The numbers refer to ePVS on one side of the brain; for analysis, the side with the higher ePVS number was used when the numbers were not bilaterally consistent (Figure 2).

Based on the median value, the CSO-ePVS degree was dichotomized into low (score: 0–1) and high (score: 2–4) levels. Additionally, based on the median value, the BG-ePVS degree was dichotomized into low (score: 1) and high (score: 2–4) levels.

Neuroradiologist blinded to the clinical data evaluated the MRI results. With a 4-week gap between the first and second image assessments, a random sample of 100 subjects was used to analyze the interrater reliability of WMH and ePVS using Cohen kappa statistics.

### 2.4. Statistical Analysis

All statistical tests were performed using SPSS version 26.0 for Microsoft Windows software. Numbers and percentages were used to represent categorical variables. Univariate analysis was performed using the chi-square test, and the variables with *p* < 0.2 were included in the logistic regression analysis. Logistic regression analysis was used to determine the best predictors for the presence of WMH and to analyze the association between the severity of ePVS and the presence of WMH. The correlation between the severities of ePVS and WMH was analyzed using Goodman–Kruskal gamma test after logistic regression showed that the severity of ePVS was significantly correlated with the presence of WMH. *p* < 0.05 was considered significant.

## 3. Results

### 3.1. Demographic Characteristics of the Study Participants

Table 1 summarizes the demographic and clinical findings of this study. A total of 887 patients, including 389 (43.8%) males and 498 (56.1%) females, were enrolled in this study. The median age of the group was 36 years (range, 16–45 years). The cohort comprised 24 (2.7%) patients with diabetes, 72 (8.1%) with hypertension and 25 (2.8%) with hyperlipidemia. Most of the patients had symptoms (94.0%), including 227 (25.6%) with headache, 177 (20.0%) with dizziness, 35 (3.9%) with vertigo, 17 (1.9%) with syncope, 27 (3.0%) with light-headedness, 70 (7.9%) with somatic symptoms, 31 (3.5%) with hearing disturbance, 21 (2.4%) with visual disturbance, 46 (5.2%) with convulsions, 21 (2.4%) with tremors, 53 (6.0%) with sleep disturbance, and 109 (12.3%) with unspecified symptoms.

### 3.2. Distribution and Severity of WMH

The prevalence of WMH among the study participants was 37.0%. Of the individuals with WMH, 96.3% (316/328) had DWMH and 20.0% (64/328) had PWMH. Most of the patients in the DWMH group had a score of 1 (79.0%, 259/328), followed by 2 (16.2%, 53/328) and 3 (1.2%, 4/328). Similarly, most of the patients in the PWMH group had a score of 1 (62.5%, 40/64), followed by 2 (25.0%, 16/64) and 3 (12.5%, 8/64) (Table 2).

### 3.3. Risk Factors of WMH

As shown in Table 3, chi-square testing indicated that age (*p* < 0.001), hypertension (*p* = 0.001), syncope (*p* = 0.044), and sleep disturbance (*p* = 0.003) were associated with DWMH. Also, age (*p* < 0.001), sex (*p* = 0.004), hypertension (*p* < 0.001), and sleep disturbance (*p* < 0.001) were associated with PWMH.

Table 4 presents the results of logistic regression analysis for the predictors of DWMH and PWMH. Age, hypertension [odds ratio (OR) = 1.915, 95% confidence interval (CI): 1.201–3.053], headache (OR = 1.450, 95% CI: 1.035–2.030), syncope (OR = 3.647, 95% CI: 1.329–10.004) and sleep disturbance (OR = 2.404, 95% CI: 1.344–4.299) were associated with the presence of DWMH. Age, sex (OR = 0.555, 95% CI: 0.324–0.950), hypertension (OR = 2.645, 95% CI: 1.370–5.107) and sleep disturbance (OR = 3.860, 95% CI: 1.855–8.032) were associated with the presence of PWMH.

### 3.4. Correlation between WMH and ePVS

As shown in Table 5, binary logistic regression analysis revealed a significant association between the severity of CSO-ePVS and the presence of DWMH (Model 1: OR = 1.828, 95% CI: 1.120–2.983, *p* = 0.016). This association was evident after controlling for confounding factors, including age, sex, vascular risk factors, and symptoms, as indicated by Model 2 (OR = 1.766, 95% CI: 1.079–2.892, *p* = 0.024) and Model 3 (OR = 1.828, 95% CI: 1.098–3.045, *p* = 0.020). The severity of BG-ePVS was associated with DWMH (Model 1: OR = 1.667, 95% CI: 1.163–2.391, *p* = 0.005). After controlling for confounding factors, this association was evident as indicated by Model 2 (OR = 1.570, 95% CI: 1.088–2.267, *p* = 0.016) and Model 3 (OR = 1.540, 95% CI: 1.059–2.239, *p* = 0.024). As shown in Table 6, no significant association was found between the severity of CSO-ePVS and the presence of PWMH in either of the three models. The severity of BG-ePVS was associated with the presence of PWMH (Model 1: OR = 3.465, 95% CI: 1.861–6.452, *p* < 0.001). This association was still evident after controlling for confounding factors, as indicated by Model 2 (OR = 3.007, 95% CI: 1.616–5.595, *p* = 0.001) and Model 3 (OR = 3.427, 95% CI: 1.802–6.520, *p* < 0.001).

The Goodman–Kruskal gamma test was used to analyze the correlation between the severities of ePVS and WMH. The severities of CSO-ePVS were positively correlated with those of DWMH (gamma = 0.263, *p* < 0.001) (Table 7). The severities of BG-ePVS were positively correlated with the severities of DWMH (gamma = 0.289, *p* < 0.001) and PWMH (gamma = 0.579, *p* < 0.001).

## 4. Discussion

In this retrospective study, we investigated WMH prevalence, risk factors, and correlation with ePVS in young clinical patients. The prevalence of WMH in our cohort was 37.0%, mostly mild. Age, hypertension, headache, syncope, and sleep disturbance were associated with the presence of DWMH while age, sex, hypertension, and sleep disturbance were associated with the presence of PWMH. The severities of CSO-ePVS were associated with the presence and severity of DWMH. The severity of BG-ePVS affected the presence and severity of DWMH and PWMH.

### 4.1. Effects of Demographic Factors on WMH

The prevalence of WMH differs between populations, wherein WMH frequently observed in older populations. Seixas et al. demonstrated that the prevalence of WMH was 95.8% among healthy individuals (age: 65.1 ± 8.2 years) [10]. Another study reported that the prevalence of WMH was 56% in healthy individuals (age: 16–78 years) [11]. The overall prevalence of WMH in the current study was 37.0%. Nonetheless, we observed a low prevalence because the median age of the current study population was 36 years. Furthermore, the 26- to 35-year-old and 36- to 45-year-old age groups experienced high degrees of DWMH and PWMH compared to the 16- to 25-year-old group. In line with previous reports, the degree of WMH increased in an age-dependent manner. Therefore, in clinical practice, WMH should be under intensive focus in older patients.

The majority of studies have concluded that sex was not correlated with WMH; while in a few studies, women presented WMH. In a cross-sectional study of middle-aged subjects, the female sex was significantly associated with DWMH [1]. The reason for the sex difference is yet to be clarified. In the Elderly at Risk (PROSPER) study, females had a high burden of WMH (both in DWMH and PWMH) [12]. This phenomenon could be attributed to decreased estrogen after menopause, allowing the brain to be more prone to hypoxia [13]. In the current study, we observed that young women had a high prevalence of PWMH indicating the correlation of female with PWMH is not related to menopause; nonetheless, a large sample size is required to confirm this finding.

Furthermore, we demonstrated that hypertension has a significant impact on the presence of DWMH and PWMH which was consistent with previous findings that hypertension is a predictor of WMH [14]. A cross-sectional cohort study indicated that structural alternations of cerebral vessels induced by high blood pressure were correlated with WMH. Decreased cerebral vessel density, decreased tortuosity and increased radius of vessels may lead to hypoperfusion which is the pathogenesis of WMH [15]. In clinical work, when WMH is incidentally detected, the radiologists and neurologists should focus on the patient’s history of hypertension.

### 4.2. Influence of Clinical Symptoms on WMH

In clinical practice, radiologists often analyze the head MRI of symptomatic patients without specific causes. Hence, we considered the effects of symptoms on WMH in this study and found that patients with, rather than without, headaches were more likely to have DWMH. Previous studies indicated that any history of severe headaches was associated with an increased risk of high volumes of total WMH, DWMH, and PWMH [16]. Thus, we hypothesized that our results are discordant due to younger age and lower severity, which might have caused headaches leading to DWMH, but not progressed to PWMH. Our study also showed that patients with syncope were likely to have DWMH. Thus, we speculated that syncope represents the poor tolerance of the brain to any changes in blood pressure indicating that vascular endothelial dysfunction leads to DWMH emergence. Therefore, in clinical practice, radiologists should be concerned about WMH when patients present headaches or dizziness.

Accumulating evidence suggests that sleep is associated with the presence of WMH. Shanshan et al. demonstrated that sleeping disorders are associated with a high burden of WMH among patients with chronic insomnia (median age = 50 years) [17]. Kristine et al. found that short sleep duration is associated with WMH in middle-aged adults [18]. Recent studies have shown that the neurotoxic metabolites are cleared during sleep, and shortened sleep duration could influence this process [19]. Consequently, accumulated neurotoxic metabolites (for example, tau protein) might form WMH [20]. The current study showed that sleep disturbance is associated with DWMH and PWMH. Therefore, sleep disorders not only affected WMH presence in the middle-aged and older people but also in the young individuals.

### 4.3. Correlation between WMH and ePVS

Almost all studies have reported that ePVS is associated with WMH. Herein, we found that the severity of BG-ePVS was associated with the presence of DWMH and PWMH in contrast with CSO-ePVS only related to the presence of DWMH. The severity of CSO-ePVS was associated with that of DWMH, while the severity of BG-ePVS was associated with those of both DWMH and PWMH. However, the underlying mechanism for the difference in the regional association between WMH and ePVS is yet unclear. Some studies suggested that BG-ePVS was caused mainly by hypertensive arteriopathy, with pathogenesis similar to WMH, while CSO-ePVS was due to cerebral amyloid angiopathy [5]. However, this phenomenon did not explicate our results. Huang et al. proposed the topological connections between DWMH and CSO-ePVS [2]. Most punctuate (<3 mm) DWMH was connected with one ePVS tube, and a large (>5 mm) DWMH could have several thick ePVS connections. This association is apparent in patients with mild WMH. In our study, this correlation was manifested due to the fact that the study population was young, and the vast majority of patients presented a mild feature.

Nevertheless, the current study has several limitations. First, we used visual rating scales to judge the severities of WMH and ePVS. Due to very little ePVS, coexisting extensive WMH, or the presence of lacunes, the reliability of this evaluation method is reduced [21]. Some special rating scales or three-dimensional automated grading scales can be used to improve the detection of CSVD [21,22]. Second, the risk factors, such as hypertension, diabetes, and hyperlipidemia, were included, while some confounding variables, such as smoking and obesity, were not included in the analysis model. Finally, the sample size of this study was small, only involved a single center, and was retrospective. In the future, multicenter prospective studies with large sample sizes will be needed to verify our findings.

## 5. Conclusions

The prevalence of WMH was 37% and was mild in young patients without specific causes. Older age, female, hypertension, headache, syncope, and sleep disturbance were associated with WMH. The severity of ePVS affected the presence and severity of WMH in the corresponding brain regions.

## Figures and Tables

**Table 1:** Basic patient information and clinical data in subjects grouped by age.

		16–25 Years (*n* = 159)	26–35 Years (*n* = 260)	36–45 Years (*n* = 468)
Sex	Male, *n* (%)	52 (32.7%)	116 (44.6%)	221 (47.2%)
Vascular risk factors	Diabetes, *n* (%)	1 (0.6%)	7 (2.6%)	16 (3.4%)
	Hypertension, *n* (%)	4 (2.5%)	20 (7.6%)	48 (12.3%)
	Hyperlipidemia, *n* (%)	2 (1.2%)	6 (2.3%)	17 (3.6%)
Symptoms	Absent, *n* (%)	6 (3.7%)	19 (7.3%)	28 (5.9%)
	Headache, *n* (%)	54 (33.9%)	54 (20.7%)	119 (25.4%)
	Dizziness, *n* (%)	19 (11.9%)	54 (20.7%)	104 (22.2%)
	Vertigo, *n* (%)	6 (3.7%)	10 (3.8%)	19 (4.0%)
	Syncope, *n* (%)	5 (3.1%)	7 (2.6%)	5 (1.0%)
	Light-headedness, *n* (%)	3 (1.8%)	9 (3.4%)	15 (3.2%)
	Somatic symptoms, *n* (%)	10 (6.2%)	30 (11.5%)	30 (6.4%)
	Hearing disturbances, *n* (%)	2 (1.2%)	9 (3.4%)	20 (4.2%)
	Visual disturbances, *n* (%)	3 (1.8%)	8 (3.0%)	10 (2.1%)
	Convulsions, *n* (%)	5 (3.1%)	21 (8.0%)	20 (4.2%)
	Tremors, *n* (%)	3 (1.8%)	6 (2.3%)	12 (2.5%)
	Sleeping disturbances, *n* (%)	5 (3.1%)	13 (5.0%)	35 (7.4%)
	Unspecified, *n* (%)	38 (23.8%)	20 (7.6%)	51 (10.8%)

**Table 2:** Imaging manifestations in subjects grouped by age.

		16–25 Years (*n* = 159)	26–35 Years (*n* = 260)	36–45 Years (*n* = 468)
DWMH	0, *n* (%)	124 (77.9%)	169 (65.0%)	278 (59.4%)
	1, *n* (%)	32 (20.1%)	74 (28.4%)	153 (32.6%)
	2, *n* (%)	3 (1.8%)	16 (6.1%)	34 (7.2%)
	3, *n* (%)	0 (0.0%)	1 (0.3%)	3 (0.6%)
PWMH	0, *n* (%)	158 (99.3%)	242 (93.0%)	423 (90.3%)
	1, *n* (%)	1 (0.6%)	9 (3.4%)	30 (6.4%)
	2, *n* (%)	0 (0.6%)	6 (2.3%)	10 (2.1%)
	3, *n* (%)	0 (0.0%)	3 (1.1%)	5 (1.0%)
CSO-ePVS score	0, *n* (%)	21 (13.2%)	0 (0.0%)	0 (0.0%)
	1, *n* (%)	128 (80.5%)	254 (97.7%)	70 (14.9%)
	2, *n* (%)	8 (5.0%)	4 (1.5%)	340 (72.6%)
	3, *n* (%)	2 (1.2%)	1 (0.4%)	55 (11.7%)
	4, *n* (%)	0 (0.0%)	1 (0.4%)	3 (0.6%)
BG-ePVS score	1, *n* (%)	154 (96.8%)	253 (97.3%)	282 (60.2%)
	2, *n* (%)	3 (3.1%)	3 (1.2%)	147 (37.1%)
	3, *n* (%)	2 (1.3%)	2 (0.8%)	8 (1.7%)
	4, *n* (%)	0 (0.0%)	2 (0.8%)	4 (0.9%)

**Table 3:** Results of the chi-square analysis of the differences in the risk factors and presence of WMH.

	DWMH		*p*-Value	PWMH		*p*-Value
	Absent (*n* = 571)	Present (*n* = 316)		Absent (*n* = 823)	Present (*n* = 64)	
Age (years)			<0.001			0.001
16–25, *n* (%)	124 (21.7)	35 (11.1)		158 (19.2)	1 (1.6)	
26–35, *n* (%)	169 (29.6)	91 (28.8)		242 (29.4)	18 (28.1)	
36–45, *n* (%)	278 (48.7)	190 (60.1)		423 (51.4)	45 (70.3)	
Male, *n* (%)	250 (43.8)	139 (44.0)	0.953	350 (42.5)	39 (60.9)	0.004
Diabetes, *n* (%)	15 (2.6)	9 (2.8)	0.846	21 (2.6)	3 (4.7)	0.539
Hypertension, *n* (%)	39 (6.8)	43 (13.6)	0.001	67 (8.1)	15 (23.4)	<0.001
Hyperlipidemia, *n* (%)	16 (2.8)	9 (2.8)	0.968	21 (2.6)	4 (6.3)	0.184
Symptoms						
Absent, *n* (%)	38 (6.7)	15 (4.7)	0.251	52 (6.3)	1 (1.6)	0.122
Headache, *n* (%)	137 (24.0)	90 (28.5)	0.142	212 (25.8)	15 (23.4)	0.682
Dizziness, *n* (%)	117 (20.5)	60 (19.0)	0.592	165 (20.0)	12 (18.8)	0.802
Vertigo, *n* (%)	24 (4.2)	11 (3.5)	0.597	31 (3.8)	4 (6.3)	0.326
Syncope, *n* (%)	7 (1.2)	10 (3.2)	0.044	17 (2.1)	0 (0.0)	0.492
Light-headedness, *n* (%)	18 (3.2)	9 (2.8)	0.801	25 (3.0)	2 (1.9)	0.999
Somatic symptoms, *n* (%)	47 (8.2)	23 (7.3)	0.614	65 (7.9)	5 (7.8)	0.981
Hearing disturbance, *n* (%)	20 (3.5)	11 (3.5)	0.987	30 (3.6)	1 (1.6)	0.603
Visual disturbance, *n* (%)	13 (2.3)	8 (2.5)	0.811	19 (2.3)	2 (3.1)	0.999
Convulsion, *n* (%)	33 (5.8)	13 (4.1)	0.284	41 (5.0)	5 (7.8)	0.489
Tremor, *n* (%)	14 (2.5)	7 (2.2)	0.824	18 (2.2)	3 (4.7)	0.401
Sleep disturbance, *n* (%)	24 (4.2)	29 (9.2)	0.003	41 (5.0)	12 (18.8)	<0.001
Unspecified, *n* (%)	79 (13.8)	30 (9.5)	0.059	107 (13.0)	2 (3.1)	0.020

**Table 4:** Logistic regression analysis for the risk factors and WMH.

	OR (95% CI)	*p*
DWMH		
Age (years)		
16–25	1 (referent)	
26–35	1.913 (1.195–3.061)	0.007
36–45	2.336 (1.514–3.605)	<0.001
Hypertension	1.915 (1.201–3.053)	0.006
Headache	1.450 (1.035–2.030)	0.031
Syncope	3.647 (1.329–10.004)	0.012
Sleep disturbance	2.404 (1.344–4.299)	0.003
Unspecified	0.885 (0.549–1.427)	0.617
PWMH		
Age		
16–25	1 (referent)	
26–35	9.852 (1.293–75.097)	0.027
36–45	12.393 (1.679–91.490)	0.014
Female	0.555 (0.324–0.950)	0.032
Hypertension	2.645 (1.370–5.107)	0.004
Hyperlipidemia	1.865 (0.591–5.883)	0.288
Absent	0.263 (0.035–1.970)	0.194
Sleep disturbance	3.860 (1.855–8.032)	<0.001

Note: Logistic regression was conducted on variables with a *p* < 0.2 in the univariate analysis.

**Table 5:** Correlation between the severity of ePVS and the presence of DWMH.

Variables	Model 1			Model 2			Model 3		
	OR	95% CI	*p*	OR	95% CI	*p*	OR	95% CI	*p*
CSO-ePVS	1.828	1.120–2.983	0.016	1.766	1.079–2.892	0.024	1.828	1.098–3.045	0.020
BG-ePVS	1.667	1.163–2.391	0.005	1.570	1.088–2.267	0.016	1.540	1.059–2.239	0.024

Note: For the logistic regression analysis, Model 1 was adjusted by age and sex; Model 2 was adjusted by age, sex, and vascular risk factors; Model 3 was adjusted by age, sex, vascular risk factors, and symptoms.

**Table 6:** Correlation between the severity of ePVS and the presence of PWMH.

Variables	Model 1			Model 2			Model 3		
	OR	95% CI	*p*	OR	95% CI	*p*	OR	95% CI	*p*
CSO-ePVS	2.551	0.910–7.153	0.075	2.074	0.794–5.421	0.137	2.067	0.787–5.431	0.141
BG-ePVS	3.465	1.861–6.452	<0.001	3.007	1.616–5.595	0.001	3.427	1.802–6.520	<0.001

Note: For logistic regression analysis, Model 1 was adjusted by age and sex; Model 2 was adjusted by age, sex, and vascular risk factors; Model 3 was adjusted by age, sex, vascular risk factors, and symptoms.

**Table 7:** Gamma coefficient analysis of the severity of ePVS and WMH scores.

	DWMH				Gamma Coefficient	*p*	PWMH				Gamma Coefficient	*p*
	0	1	2	3			0	1	2	3		
CSO-ePVS					0.263	<0.001						
Low	333 (70.4)	117 (24.7)	23 (4.9)	0 (0.0)								
High	238 (57.5)	142 (34.3)	30 (7.2)	4 (1.0)								
BG-ePVS					0.289	<0.001					0.579	<0.001
Low	467 (67.8)	183 (26.6)	37 (5.4)	2 (0.3)			657 (95.4)	18 (2.6)	9 (1.3)	5 (0.7)		
High	60 (52.5)	46 (38.4)	11 (8.1)	1 (1.0)			166 (83.8)	22 (11.1)	7 (3.5)	3 (1.5)		

Note: Correlation between the severities of ePVS and WMH was analyzed after logistic regression showed that the severity of ePVS was significantly correlated with the presence of WMH.

## Data Availability

Data are available on request from the corresponding author.
